# Efficacy of Anti-Vascular Endothelial Growth Factor Treatment in Neovascular Age-Related Macular Degeneration and Systemic Cardiovascular Risk Factors

**DOI:** 10.3390/jcm10194595

**Published:** 2021-10-06

**Authors:** Joanna Łądkowska, Maciej Gawęcki, Marek Szołkiewicz

**Affiliations:** 1 Department of Ophthalmology, Pomeranian Hospitals, 84-200 Wejherowo, Poland; j_ladkowska@wp.pl; 2 Dobry Wzrok Ophthalmological Clinic, 80-280 Gdansk, Poland; 3 Department of Cardiology and Interventional Angiology, Kashubian Center for Heart and Vascular Diseases, Pomeranian Hospitals, 84-200 Wejherowo, Poland; e.mars@wp.pl

**Keywords:** age-related macular degeneration, anti–vascular endothelial growth factor, cardiovascular risk factors, arterial hypertension

## Abstract

This study evaluates whether the presence of cardiovascular risk factors (CRFs) affects functional and morphological responses to anti–vascular endothelial growth factor (VEGF) therapy in patients with neovascular age-related macular degeneration (nAMD). Retrospective analysis included 98 treatment-naïve eyes followed for at least 12 months. Patients received intravitreal injections of ranibizumab or aflibercept with the dosage and regimen set according to each manufacturer’s recommendations for their product. Parameters evaluated at each follow-up visit included best-corrected visual acuity and central retinal thickness. Additionally, the presence of the following CRFs was evaluated: male sex, age of older than 70 years, history of current or past smoking, systemic arterial hypertension, diabetes mellitus, total hypercholesterolemia, low-density lipoprotein hypercholesterolemia, high-density lipoprotein concentration of 45 mg/dL or less, atherogenic dyslipidemia, family history of cardiovascular disease, and chronic kidney disease. A statistically significant better letter gain in visual acuity (*p* = 0.012) and greater percentage of responders (*p* = 0.035)—that is patients in whom best corrected visual acuity was stabilized or improved at 12 months—were noted among patients without a diagnosis of arterial hypertension. A statistically significant better mean visual improvement was also achieved in patients with higher total cholesterol plasma levels *(p* = 0.004), but this finding was not reflected in the significantly higher percentage of responders. The presence of remaining analyzed risk factors did not substantially affect the results of treatment. Systemic arterial hypertension is an independent factor leading to a poor functional outcome following anti-VEGF therapy in patients with nAMD. Effects of anti-VEGF treatment in patients with high total cholesterol levels should be analyzed in further research.

## 1. Introduction

Age-related macular degeneration (AMD) is a common retinal disorder typically affecting elderly individuals and one of the leading causes of blindness worldwide in those older than 50 years of age [1,2,3]. The introduction of intravitreal injections of anti-vascular endothelial growth factor (anti-VEGF) agents has led to a reduction of 50% of patients with the neovascular form of AMD (nAMD) who end up blind; however, the group of patients, whose vision deteriorates despite receiving treatment, remains of great concern for clinicians [4]. It should be noted that, in a large percentage of patients, best-corrected visual acuity (BCVA) declines over the years despite a rigorous treatment regime and good compliance [5,6,7].

Factors influencing the outcome of anti-VEGF treatment of nAMD have been analyzed in many clinical studies to date [8,9,10]. Traditionally, the attention of researchers has been directed toward the efficacy of a specific agent or retinal morphological biomarkers at the beginning of treatment, and the analysis of systemic factors affecting the results of intravitreal therapy has not been of interest to researchers around the world. To the best of our knowledge, only a small number of papers exist that directly seek to elucidate this relationship, including such risk factors as smoking, body mass index (BMI), and systemic hypertension [11,12,13,14,15,16,17,18,19,20]. As numerous studies prove, both cardiovascular disorders and AMD show similarities in their etiopathogenesis [21,22,23]. According to the literature, the prevalence of AMD is more frequent in patients with cardiovascular diseases [24,25]. Moreover, patients with AMD are at greater higher risk for stroke and cardiovascular mortality [26,27,28]. On the other hand, cardiovascular disorders are frequently listed as risk factors for AMD development [29,30,31]. These facts inspired us to analyze the relationship between cardiovascular risk factors (CRFs) and the effects of anti-VEGF treatment in patients with nAMD.

## 2. Materials and Methods

All procedures performed in this study were conducted in accordance with the ethical standards of the institutional research committee and the 1964 Declaration of Helsinki. The study was approved by the local bioethical board (Komisja Bioetyczna at OIL in Gdańsk, approval no. KB-29/18).

**Figure 1:**
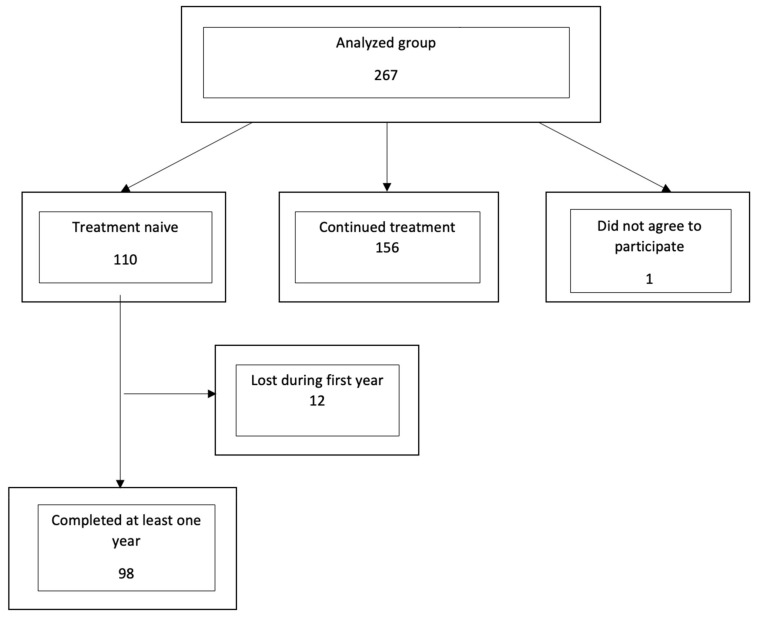
Flowchart for the selection of eyes included in the study.

The retrospective analysis included 267 eyes of consecutive patients who began treatment for nAMD within the Drug Program of Treatment of nAMD (DP) in the ophthalmological ward and outpatient clinic of Wejherowo Hospital between 2015 and 2018. Written consent for inclusion in the study was obtained in 266 cases. Only treatment-naïve cases were selected from the whole group and included in the study. Patients who joined the program after undergoing treatment previously were excluded. In cases where both eyes of the same patient were treated, only one eye was randomly selected for the study. The study inclusion criteria limited the number of study eyes (patients) to 110, and 98 ultimately completed at least one year of follow-up. The study group consisted of 67 women and 31 men with a mean age of 76.5 ± 7.65 years. The flowchart for patient recruitment into the study is presented in Figure 1.

All study participants were diagnosed and followed up with according to the rules of the DP. A diagnosis of nAMD was established on the basis of the following assessments: BCVA, fluorescein angiography (FA), and spectral-domain optical coherence tomography (SD-OCT). BCVA was assessed on standard Early Treatment Diabetic Retinopathy Study 4-m charts, FA was performed using the Visucam NM/FA system (Carl Zeiss, Oberkochen, Germany), and SD-OCT was performed using the Cirrus 5000 system (Carl Zeiss). The most important inclusion criteria for DP were a BCVA between 0.2 and 0.8 on the Snellen chart (i.e., ETDRS letter score of between 50 and 80) and the presence of active subfoveal neovascularization confirmed by the FA and SD-OCT findings. 

Patients were treated with either ranibizumab (Lucentis; Genentech, San Francisco, CA, USA) or aflibercept (Eylea; Regeneron, Tarrytown, NY, USA) according to the dosing regimen recommended by the manufacturer of each product. Ranibizumab was administered in 32 cases, and aflibercept was given in 66 cases. Patients were randomized to receive either aflibercept or ranibizumab, however, at the moment of introduction of DP, only aflibercept was reimbursed. Ranibizumab was introduced to DP later, and that explains the difference in numbers of aflibercept and ranibizumab injections performed in the study.

Treatment with ranibizumab was initiated with one injection given per month until maximum visual acuity was achieved and/or there were no signs of disease activity during ophthalmological examination and SD-OCT. Afterward, the drug was administered in a pro re nata fashion.

Treatment with aflibercept was initiated with one injection given per month for five consecutive doses, then one injection given every two months until the end of the first year. Subsequently, the drug was administered in a pro re nata fashion. The retreatment criteria included any loss in BCVA and the presence of disease activity on SD-OCT, which was suggested by any increase in central retinal thickness (CRT), any increase in the amount of pigment epithelial detachment (PED), onset of subretinal or intraretinal fluid, or hemorrhage. Measurements of CRT using the Carl Zeiss Cirrus 5000 system refer to the mean retinal thickness within the central area of the retina measuring 1 mm in diameter. 

BCVA assessments and SD-OCT imaging were performed for each participant every month. Statistical analysis included baseline, three-month, and 12-month follow-up results.

During their baseline examination, each of the patients included in the study was interviewed according to the presence of selected CRFs. The following factors were analyzed: male sex, age of greater than 70 years, history of current or past smoking (not applicable if patient had not smoked for at least 20 years), systemic arterial hypertension (AH) (BP ≥ 140 mmHg or intake of hypotensive medications), diabetes mellitus (blood glucose concentration > 126 mg/dL or intake of hypoglycemic medications), total hypercholesterolemia (total cholesterol [TCH] ≥ 190 mg/dL), low-density lipoprotein (LDL) hypercholesterolemia (LDL ≥ 115 mg/dL or intake of cholesterol-lowering medications), high-density lipoprotein (HDL) concentration of 45 mg/dL or less, atherogenic dyslipidemia (triglyceride level > 150 mg/dL and HDL ≤ 45 mg/dL or intake of lipid-lowering medications), family history of cardiovascular disease (e.g., stroke, myocardial infarction, or peripheral atherosclerosis in a close family member younger than 60 years of age), and chronic kidney disease (CHKD) as evaluated by the estimated glomerular filtration rate (<60 mL/min/1.73 m^2^).

Apart from using patients’ medical history, CRFs were evaluated in laboratory tests conducted before initiation of anti-VEGF treatment for all patients in a certified hospital laboratory; tests included the assessment of plasma levels of total TCH, HDL, LDL, triglycerides, creatinine, and glucose. The estimated glomerular filtration was calculated using the Chronic Kidney Disease Epidemiology Collaboration formula [32]. Blood pressure was measured before and throughout the study. 

All of the patients had anthropometric measurements collected during their baseline examination, including their weight (kg) and height (cm), which were used to calculate the body mass index (BMI). A BMI of at least 30 kg/m^2^ was considered a CRF. Additionally, the circumferences of the waist and hips were measured using the World Health Organization (WHO) data-gathering protocol, where the waist circumference was measured at the midpoint between the lower margin of the last palpable rib and the top of the iliac crest, and the hip circumference was measured around the widest portion of the buttocks. Results were used to calculate the waist-to-hip ratio (WHR); abdominal obesity was recognized when the WHR value was more than 1 in men or 0.85 in women, according to WHO recommendations [33].

The distribution of analyzed CRFs in the study group is detailed in Table 1.

The morphological response was considered good (i.e., the patient was responsive to treatment) if any reduction in CRT after 12 months of treatment was noted. Conversely, the patient was considered a non-responder if their CRT increased after 12 months of treatment.

The response to anti-VEGF treatment was defined as functionally good if the BCVA was stabilized or improved at 12 months (and the patient was deemed a responder to treatment). Non-responders were those who showed a decrease in BCVA at the end of the follow-up period.

### Statistical Analysis

The analysis of the gathered data included the following results and correlations:

- Changes in BCVA and CRT at 3 and 12 months of treatment in the whole study group.
- Mean changes in BCVA and CRT in the subgroups of patients with and without specific CRF.
- Proportions of responders and non-responders (functional and morphological) according to the presence of each specific CRF.
- Correlation between the changes in BCVA and CRT at 12 months and the number of coexisting CRFs.

The relationship between the presence of specific CRFs and the response to treatment was evaluated in two ways. First, the changes in BCVA and CRT were compared between patients with and without a specific CRF. Second, the percentages of responders and non-responders were calculated and compared between these groups.

Statistical analysis in this study had to take into account not only mean improvements in BCVA and CRT but also the proportions of responders and non-responders. Statistical significance of the measured changes in mean BCVA and CRT values without a statistically significant difference between the percentages of responders and non-responders may suggest a substantial dispersion of individual results and not reflect the existence of a true trend in the whole study cohort

Statistical analysis was performed using the Statistica version 10.0 software program (StatSoft, Tulsa, OK, USA). Quality variables were presented with the frequency distribution.

For quotative variables, arithmetic mean, standard variation, and minimum and maximum values were calculated. The normalcy of distribution was evaluated with the Shapiro–Wilk test. 

As the variables did not fulfill the criteria for the use of parametric methods, nonparametric tests were used to verify statistical hypotheses. The following tests were used for analysis: ANOVA Friedman with the post-hoc Dunn–Bonferroni test (analysis of variance with correction for multiple comparisons), chi-squared test (with the Yates amendment for small samples), Mann–Whitney U test, and Spearman’s rank correlation test. The results were regarded as statistically significant when *p* < 0.05.

## 3. Results

The mean number of intravitreal injections administered per eye in the whole group during 12 months of follow-up was 6.65 ± 1.41 injections, with a median of seven injections, minimum of three injections, and maximum of nine injections, respectively. The mean numbers of injections per eye during the study period were 5.7 in those receiving ranibizumab and 7.54 in those receiving aflibercept. BCVA was improved by a significant mean result of 4.57 ± 14.03 letters at 12 months, while CRT was reduced by a significant mean value of 130.76 ± 173.59 μm; exact data on these changes are presented in Table 2. Functional and morphological improvements occurred in 75.51% and 82.65% of patients, respectively (Table 3).

According to our results, there were no significant differences in the functional or morphological outcome between the use of aflibercept or ranibizumab. We noted mean BCVA improvement of 5.09 ± 13.67 letters in case of aflibercept versus 3.5 ± 15.77 for ranibizumab (*p* = 0.753) and CRT reduction of mean 148.5 ± 198.49 μm in case of aflibercept versus 94.16 ± 98.11 μm for ranibizumab (*p* = 0.55). 

Data concerning the correlation of the presence of specific CRFs and the efficacy of treatment are provided in Table 4 and Table 5. Statistically significantly greater improvements in BCVA were achieved in patients without a diagnosis of AH (10.07 ± 11.91 vs. 2.26 ± 14.7; *p* = 0.012); this result was also true with respect to the higher percentage of responders to anti-VEGF treatment (89.66% vs. 69.57%; *p* = 0.035). Meanwhile, a statistically significant letter gain improvement was also confirmed in patients with higher TCH levels (7.78 ± 14.56 vs. 1.23 ± 13.43 mg/dL; *p* = 0.004), but this result was not reflected in the substantially higher percentage of responders (82.00% vs. 68.75%; *p* = 0.127). An analogous situation was noted in patients without CHKD; however, this result bordered on statistical significance (*p* = 0.051) and was not reflected in a difference between responders and non-responders. There was a tendency toward better mean CRT reduction in patients with LDL hypercholesterolemia, and this result also bordered on statistical significance (*p* = 0.048) and was not apparent in the ratio of responders to non-responders. Neither response group experienced better functional improvement over the other.

As patients with AH generally tend to be older patients free of that disease, we performed additional comparison of AH patients versus non-AH patients in reference to their age to assess the bias of age-factor. Analysis revealed mean age in AH group is 77.20 ± 7.69 versus 75.07 ± 7.47 in non-AH group. The difference is not significant in *t*-test with *p* = 0.22.

Correlation between an increasing number of CRFs and changes in BCVA and CRT were not found; however, there was a tendency toward worse functional improvement in patients with greater numbers of CRFs (*p* = 0.052). Spearman’s rank correlation coefficient values are provided in Table 6.

## 4. Discussion

In this section, we present discussion of the results of our study according to each risk factor analyzed. 

### 4.1. Systemic Hypertension

The relationship between elevated systemic blood pressure and AMD has been analyzed in clinical trials; however, research has concentrated on the evaluation of AH as a risk factor for the development of AMD, not the effects of anti-VEGF treatment. Orthostatic blood pressure behavior (rise after assuming the upright position) was associated with an increased risk of AMD in a recent study [34]. A significant difference between systolic and diastolic pressure values was linked to a greater risk of late AMD in the ALIENOR study [35]. Higher values of systolic blood pressure were also correlated with a higher prevalence of late AMD in the Women’s Health Initiative Sight Exam ancillary study [36]. In the Beaver Dam Study, it was also proven that the use of blood pressure–lowering medications, especially beta-blockers, is associated with a higher incidence of nAMD [37]. Other research has argued that the presence of systemic hypertension results in decreased choroidal flow in non-exudative forms of AMD and impaired choroidal perfusion results in insufficient elimination of degradation products from the RPE and the formation of drusen [38]. Moreover, reduced blood flow in the choroid stimulates hypoxia and promotes VEGF upregulation and neovascularization [39]. That mechanism in later exudative stages could explain the insufficient effect of VEGF blockers in nAMD population with accompanying AH. Nevertheless, a direct relationship between the effects of nAMD treatment and the presence of AH has been investigated in only a few trials to date.

One study by Piermarocchi et al. showed that complement factor H risk alleles, smoking history, and AH each independently influenced the patient’s response to ranibizumab treatment of nAMD, with worse 12-month BCVA outcomes (*p* = 0.036, *p* = 0.037, and *p* = 0.043, respectively) [11]. These authors recorded a mean improvement of 3.0 ± 8.1 letters in patients without AH versus that of −0.6 ± 9.1 in patients affected by AH.

Better functional results in patients without AH were also reported by Menger et al., [19] who documented BCVA changes at 24 months of treatment of −0.01 logMAR in patients on hypotensive drugs versus +0.21 logMAR in patients without AH (*p* = 0.045).

On the other hand, Zhao et al. and van Asten et al. did not find a correlation between the presence of AH and poor outcomes with intravitreal therapy among nAMD patients [12,16].

Our results are consistent with data obtained in the studies by Piermarocchi et al. and Menger et al. In our cohort, the absence of AH was related to significantly better visual gains (10.07 vs. 2.26 letters) and a greater percentage of functional responders (89.66% versus 69.57%); however, reductions in CRT were similar between patients with and without AH. In all of our patients, AH was well-controlled during the study period; however, all study participants experienced systemic blood pressure alterations before their inclusion in the study. 

It can be speculated that the presence of AH prior to application of anti-VEGF therapy decreases the potential for BCVA improvement. It might be true that these patients had already worse BCVA compared to the cohort without AH before the development of macular neovascularization, which would explain the worse functional improvement documented despite relatively the good morphological response to anti-VEGF. As proved by research quoted earlier [38,39] AH impairs choroidal flow and nutrition of the RPE and photoreceptors. That theoretically could result in loss of photoreceptors and visual impairment, even in the absence of neovascularization. In our study both, AH and non-AH groups achieved relatively good morphological results (significant reduction of CRT), however functional improvement was much better in cohort free of AH. This fact could also be explained by the lack of potential for improvement due to atrophic retinal alterations preceding the development of the choroidal neovascularization in patients with AH. 

Our results suggest, that good control of blood pressure before the onset of nAMD might improve the results of anti-VEGF treatment. Nevertheless, this concept should be confirmed by further studies. Further research is also needed on the impact of the use of different hypotensive drugs on visual outcome during the treatment. 

### 4.2. Plasma Lipids

Results pertaining to the relationship between the efficacy of AMD treatment and plasma lipid levels did not show any unequivocal positive or negative trend. In our cohort, lower LDL levels were associated with a better morphological reaction to anti-VEGF treatment (i.e., larger CRT reduction); however, this result bordered on statistical significance. Moreover, we did not find a correlation between LDL level and functional improvement in our patients. On the other hand, a high TCH level was related to significantly better mean letter gains with strong statistical significance (*p* = 0.004), but not a greater percentage of responders. In other words, there were cases with high TCH levels and spectacular improvements in BCVA, but such was not the rule for the whole group of patients with high TCH. Besides, morphological improvements in patients with high plasma TCH levels were not better than those in patients with lower levels of TCH. Available epidemiological studies suggest there is a lower risk for the early stages of AMD in cases with high TCH levels [40,41,42]. On the other hand, a relationship between TCH and its fractions levels and the late stages of AMD has not yet been clearly established [43,44]. Moreover, to our knowledge, no relationship between the level of plasma cholesterol and the response to anti-VEGF treatment in AMD has been established so far.

As we know from the literature, the relationship between plasma lipids and AMD is not straightforward. A large meta-analysis of available trials analyzing the risk of AMD and CRFs proved that high HDL levels are associated with a greater risk of progression to AMD, while high LDL and TCH levels play a protective role against such [45,46]. In other words, a systemic factor commonly accepted as protective against atherosclerosis, i.e., a high level of HDL, does not protect against the development of AMD [47,48,49]. There exists a concept that high HDL levels in the macula, which is constantly exposed to light and, consequently, oxygen stress, result in the production of high levels of reactive oxygen species. These species react with HDL and are converted into pro-inflammatory and pro-oxidant products, which impair cholesterol elimination and promote LDL oxygenation in the retinal pigment epithelium (RPE) [50,51]. In this way, protective HDL properties would be outweighed by the local inflammatory reactions in a manner that leads to an accumulation of residual products in the RPE [52,53]. In the light of these data, HDL appears to be a new target in treatment strategies for AMD [54]. In our study, we did not find any significant relationship between the response to anti-VEGF therapy and HDL levels in patient blood, nor was there such a correlation in reference to triglyceride levels. Interestingly, patients with lower LDL levels tended to achieve better mean CRT reductions after anti-VEGF therapy, but the percentage of responders was not higher here relative to among patients with higher LDL levels. The BCVA improvement was also not significantly greater in patients with lower LDL levels. 

Some research suggests that lowering fat levels may improve advanced non-exudative forms of AMD, such as drusenoid epithelial retinal detachments [55]. In a multicenter study of 23 patients treated with 80 mg of atorvastatin daily, Vavvas et al. noted a regression of drusenoid deposits and visual improvement by a mean of 3.3 letters in 10 subjects. Nevertheless, this kind of reasoning cannot be applied to exudative forms of AMD. 

A positive relationship between high TCH levels and visual gain following anti-VEGF treatment in selected patients is an interesting phenomenon but one that needs to be confirmed in a larger sample. No matter what future research shows, it is hard to believe that such data will be extrapolated into clinical recommendations.

### 4.3. CHKD 

Patients with CHKD tend to have poorer visual gains after anti-VEGF treatment; however, this relationship borders on statistical significance (*p* = 0.051). Besides, the difference between the percentages of responders and non-responders in this group does not depend upon the presence of CHKD as a risk factor. It has to be emphasized that the presence of CHKP has not been analyzed thus far as a risk factor for poor response in the treatment of nAMD, so we are unable to correlate our findings with other data.

### 4.4. Other CRFs

We did not find any correlation between the remaining risk factors and the effects of anti-VEGF therapy. Neither age, sex, smoking, obesity, diabetes, dyslipidemia, nor family history correlated significantly with the outcome of therapy. These findings remain partly consistent with those of other authors; however, it has to be emphasized that, quite often, the results of studies are contradictory. We present a summary of available studies linking CRFs to the efficacy of nAMD treatment in Table 7. Most of the identified studies did not confirm a relationship between age and the effect of anti-VEGF treatment of nAMD. Only Shah et al. and van Asten et al. reported better responses to anti-VEGF treatment in younger patients [14,16], while Bek et al. suggested a better morphological response occurred in older patients. Again, most of the selected studies do not show a relationship between sex and anti-VEGF therapy, and some that explore such a correlation present contradictory conclusions [13,14,15]. There exists just one study by van Asten et al. that reports a higher risk for non-responders in patients with diabetes [16]. Smoking is an obvious risk factor for AMD and cardiovascular diseases; however, studies that prove a correlation between tobacco intake and the results of anti-VEGF treatment of nAMD offer inconsistent results [11,14,19,20,21]. Only one study by Zhao et al. documented a relationship between nAMD treatment and BMI [12]. Paradoxically, a higher BMI was linked to a better response to treatment; however, the difference between BMI values among responders and poor responders was not large (26.4 ± 0.4 vs. 24.9 ± 0.5 kg/m^2^) if we consider BMI as a range; both values remain in the upper normal range of the BMI scale.

### 4.5. Number of Risk Factors

An increasing number of risk factors tended to correlate with poorer BCVA improvement; however, these result only bordered on statistical significance.

### 4.6. Limitations of the Study 

Choosing the ‘right points’ to evaluate the effects of treatment for a disease such as nAMD (which requires numerous retreatments and a long course with improvements and recurrences) is challenging. In that sense, a 12-month observation period might seem to be a relatively short period of time. On the other hand, some patients who comply with rigorous treatment regimens still lose visual acuity, even in a relatively short period of time. We believe that a 12-month period is long enough to assess factors that influence the final outcome of therapy.

We also realize that the analyzed material is relatively small for assessing as many risk factors as we did; however, statistical significance was possibly achieved in certain areas. Still, a multivariate analysis was not possible due to our relatively small sample in relation to the number of risk factors analyzed. Collecting further material will continue to support our conclusions.

## 5. Conclusions

Systemic AH is an independent factor leading to a poor functional outcome following anti-VEGF treatment of nAMD. Other cardiovascular risk factors—such as age; sex; smoking; obesity; diabetes; high LDL, HDL, and TG plasma levels; dyslipidemia; and family history—do not have a strong influence on the effects of such treatment. Further research is needed to analyze the effects of anti-VEGF treatment in patients with high TCH levels.

## Figures and Tables

**Table 1:** Distribution of risk factors in the study population.

Risk Factor	No.	%
Age ≥ 70 years	77	78.57
Male sex	31	31.63
Smoking	32	32.65
Systemic arterial hypertension	69	70.41
Diabetes	28	28.57
BMI > 30 kg/m^2^	36	36.73
WHR > 1 for M and > 0.85 for F	61	62.24
Total hypercholesterolemia	50	51.02
Hypertriglyceridemia (triglyceride level ≥ 150 mg/dL)	21	21.43
LDL hypercholesterolemia	77	78.57
HDL ≤ 45 mg/dL	12	12.24
Dyslipidemia	9	9.18
CHKD	28	28.57
Family history	17	17.35

Abbreviations: BMI, body mass index; CHKD, chronic kidney disease; HDL, high-density lipoprotein; LDL, low-density lipoprotein; TCH, total cholesterol; WHR, waist-to-hip ratio.

**Table 2:** Effects of anti-VEGF treatment at 12 months.

Variable	Before Treatment	At 3 Months	At 12 Months	*p*-Value
BCVA (ETDRS letters), mean	77.5 ± 14.09	81.08 ± 12.18	82.07 ± 13.9	0.000
CRT (μm)	434.6 ± 214.33	340.23 ± 185.67	303.85 ± 129.92	0.000

Abbreviations: BCVA, best-corrected visual acuity; CRT, central retinal thickness; ETDRS, Early Treatment Diabetic Retinopathy Study; VEGF, vascular endothelial growth factor.

**Table 3:** Stratification of responders and non-responders according to changes in BCVA and CRT at 12 months.

Parameter	Improved or Stable	%	Worsened	%
BCVA change	74	75.51	24	24.49
CRT change	81	82.65	17	17.35

Abbreviations: BCVA, best-corrected visual acuity; CRT, central retinal thickness.

**Table 4:** The change of BCVA and CRT at 12 months according to presence or absence of a specific risk factor. *P* values refer to the statistical significance of the difference in that change between patients with a specific risk factor present and absent.

Risk Factor	RF Present		RF Absent		*p* Value	RF Present		RF Absent		*p* Value
	BCVA Baseline Mean	BCVA Mean Change (+)	BCVA Baseline Mean	BCVA Mean Change (+)		CRT Baseline Mean	CRT Mean Change (−)	CRT Baseline Mean	CRT Mean Change (−)	
Age ≥ 70	62.94 ± 14.30	4.39 ± 13.37	60.86 ± 13.57	5.24 ± 17.76	0.307	435.95 ± 219.44	128.49 ± 174.69	429.67 ± 199.46	139.05 ± 173.49	0.778
Male gender (males vs. females)	59.70 ± 12.50	M 6.52 ± 10.98	63.78 ± 14.70	F 3.67 ± 15.63	0.328	587.0 ± 168.29	M 156.26 ± 178.51	452.50 ± 188.26	F 118.96 ± 151.73	0.541
Smoking	64.97 ± 11.98	2.25 ± 16.02	61.28 ± 14.97	5.70 ± 13.42	0.515	433.97 ± 209.64	116.94 ± 162.17	434.91 ± 218.16	137.46 ± 179.68	0.625
Systemic hypertension	63.00 ± 13.55	2.26 ± 14.7	61.28 ± 15.54	10.07 ± 11.91	0.012	332.5 ± 181.75	120.91 ± 157.59	429.55 ± 275.58	154.17 ± 208.01	0.770
Diabetes	63.93 ± 13.05	3.39 ± 10.00	61.91 ± 14.56	5.04 ± 15.77	0.227	411.21 ± 178.45	113.82 ± 165.96	443.96 ± 227.61	137.53 ± 177.26	0.514
Obesity BMI > 30	63.78 ± 12.83	5.42 ± 13.67	61.74 ± 14.85	3.11 ± 15.48	0.526	414.83 ± 181.80	129.50 ± 178.56	446.08 ± 231.77	131.48 ± 172.11	0.979
Obesity WHR > 1 for M and >0.85 for F	62.92 ± 13.99	4.77 ± 13.98	61.78 ± 14.47	4.24 ± 15.07	0.905	394.08 ± 185.22	106.26 ± 155.20	501.40 ± 243.37	171.14 ± 195.84	0.051
Total hypercholesterolemia TCH ≥190 mg/dL	60.44 ± 14.68	7.78 ± 14.56	64.62 ± 13.30	1.23 ± 13.43	0.004	446.36 ± 232.18	140.52 ± 149.58	422.35 ± 195.72	121.69 ± 145.31	0.722
TG ≥ 150 mg/dL	62.48 ± 15.35	4.38 ± 10.97	62.49 ± 13.86	4.62 ± 15.18	0.553	468.38 ± 189.39	176.38 ± 169.26	425.39 ± 220.88	118.31 ± 173.75	0.060
Hypercholesterolemia LDL ≥115 mg/dL	63.82 ± 13.06	3.65 ± 13.93	57.62 ± 16.91	7.95 ± 15.57	0.553	419.45 ± 206.37	113.88 ± 169.48	490.14 ± 238.39	192.62 ± 178.58	0.048
HDL ≤ 45 mg/dL	63.00 ± 14.27	−0.92 ± 19.39	62.42 ± 14.17	5.34 ± 13.44	0.383	353.92 ± 110.40	95.67 ± 103.91	445.86 ± 223.15	135.65 ± 181.09	0.766
Dyslipidemia	61.33 ± 15.18	5.22 ± 8.77	62.60 ± 14.08	4.51 ± 14.81	0.922	413.00 ± 135.08	135.11 ± 111.10	436.79 ± 221.19	131.60 ± 178.39	0.777
Chronic kidney disease	63.54 ± 13.70	2.04 ± 7.73	62.07 ± 14.34	5.59 ± 16.17	0.051	425.50 ± 179.38	129/14 ± 126.60	438.24 ± 227.90	131.40 ± 189.97	0.524
Family history	65.65 ± 14.29	−0.53 ± 18.71	61.83 ± 14.07	5.64 ± 13.12	0.120	356.82 ± 147.59	117.00 ± 139.12	450.92 ± 223.13	133.64 ± 180.60	0.778

BCVA—best corrected visual acuity, CRT—central retinal thickness, M—males F—females, WHR—waist to hip ratio HDL—high density lipoprotein cholesterol LDL—low density lipoprotein cholesterol TCH—total cholesterol RF—risk factor.

**Table 5:** Difference between percentage of responders to treatment at 12 months according to the presence or absence of a specific risk factor. *p* values refer to statistical significance of that difference between patients with risk factor present versus patients with risk factor absent.

Risk Factor	Responders BCVA (%)		*p* Value	Responders CRT (%)		*p* Value
	RF Present	RF Absent		RF Present	RF Absent	
Age ≥ 70	83.87	80.95	0.513	84.42	90.48	0.725
Male gender (males vs. females)	M 83.87	F 71.64	0.19	M 90.32	F 79.10	0.173
Smoking	68.75	78.79	0.278	78.13	84.85	0.410
Systemic hypertension	69.57	89.66	0.035	81.16	86.21	0.547
Diabetes	67.86	78.57	0.265	82.14	82.86	0.833
Obesity BMI > 30	69.44	79.03	0.287	83.33	82.26	0.892
Obesity WHR > 1 for M and >0.85 for F	73.77	78.38	0.607	77.05	91.89	0.060
Total hypercholesterolemia TCH ≥190 mg/dL	82.00	68.75	0.127	84.00	81.25	0.719
TG ≥ 150 mg/dL	71.43	76.62	0.624	90.48	80.52	0.457
Hypercholesterolemia LDL ≥115 mg/dL	75.32	76.19	0.935	81.82	85.71	0.926
HDL ≤ 45 mg/dL	66.67	76.74	0.688	83.33	82.56	0.733
Dyslipidemia	77.78	75.28	0.810	77.78	83.15	0.955
Chronic kidney disease	75.00	75.71	0.941	89.29	80.0	0.423
Family history	58.82	79.01	0.147	76.47	83.95	0.698

BCVA—best corrected visual acuity, CRT—central retinal thickness, CRF—cardiovascular risk factor M—males F—females, WHR—waist to hip ratio HDL—high density lipoprotein cholesterol LDL—low density lipoprotein cholesterol TCH—total cholesterol RF—risk factor.

**Table 6:** Number of risk factors and changes in BCVA and CRT at 12 months (Spearman’s rank correlation).

Pair of Variables	R	t(N-2)	*p*-Value
No. of risk factors and change in BCVA	−0.20	−1.97	0.052
No. of risk factors and change in CRT	0.01	0.05	0.959

Abbreviations: BCVA, best-corrected visual acuity; CRT, central retinal thickness; No., number; R, Spearman’s coefficient.

**Table 7:** Presence or absence of statistically significant correlations between the presence of selected systemic risk factors and the response to anti-VEGF treatment of nAMD.

Study	Age	Sex	DM	HA	Smoking	BMI/ Obesity	Plasma Lipids	CHKD
Present study	No	No	No	Yes (worse VA gains)	No	No	No	No
Bek et al., 2018 [13]	Yes (larger CRT reduction in older age)	Yes (smaller CRT reduction in males)	NA	NA	No	NA	NA	NA
Shah et al., 2016 [14]	Yes (better VA gains in younger patients)	Yes (better VA gains in males)	NA	NA	Yes (better final VA)	NA	NA	NA
Piermarocchi et al., 2014 [11]	NA	NA	NA	Yes (worse VA gains)	Yes (worse VA gains)	No	NA	NA
Guber et al., 2014 [15]	No	Yes (better CRT reduction in males)	NA	NA	NA	NA	NA	NA
Van Asten et al., 2014 [16]	Yes (increasing risk for Non-responders in older age)	No	Yes (risk for Non-responders)	No	No	No	NA	NA
Zhao et al., 2013 [12]	No	No	No	No	No	Yes (BMI higher in responders)	NA	NA
Krebs et al., 2013 [18]	No	No	NA	NA	NA	NA	NA	NA
Lee et al., 2013 [19]	No	No	No	No	Yes (lower VA gains in current smokers)	No	No	NA
Menger et al., 2012 [20]	No	NA	NA	Yes (worse fiNAl VA)	Yes (worse final VA)	NA	NA	NA
Inglehearn et al., 2012 [21]	No	No	NA	NA	Yes (better VA gains in smokers and ex-smokers)	NA	NA	NA

Abbreviations: BMI, body mass index; CHKD, chronic kidney disease; CRT, central retinal thickness; DM, diabetes mellitus; NA, not analyzed; VA, visual acuity.

## Data Availability

Data are available from the corresponding author upon request.
